# New Emergence of the Novel Pestivirus Linda Virus in a Pig Farm in Carinthia, Austria

**DOI:** 10.3390/v14020326

**Published:** 2022-02-05

**Authors:** Alexandra Kiesler, Lukas Schwarz, Christiane Riedel, Sandra Högler, René Brunthaler, Katharina Dimmel, Angelika Auer, Marianne Zaruba, Marlene Mötz, Kerstin Seitz, Andrea Ladinig, Benjamin Lamp, Till Rümenapf

**Affiliations:** 1 Department for Pathobiology, Institute of Virology, University of Veterinary Medicine, Veterinaerplatz 1, 1210 Vienna, Austria; Alexandra.Kiesler@vetmeduni.ac.at (A.K.); Christiane.Riedel@vetmeduni.ac.at (C.R.); Katharina.Dimmel@vetmeduni.ac.at (K.D.); Angelika.Auer@vetmeduni.ac.at (A.A.); Marianne.Zaruba@vetmeduni.ac.at (M.Z.); Marlene.Moetz@vetmeduni.ac.at (M.M.); Kerstin.Seitz@vetmeduni.ac.at (K.S.); 2 University Clinic for Swine, Department for Farm Animals and Veterinary Public Health, University of Veterinary Medicine, Veterinaerplatz 1, 1210 Vienna, Austria; Lukas.Schwarz@vetmeduni.ac.at (L.S.); Andrea.Ladinig@vetmeduni.ac.at (A.L.); 3 Department for Pathobiology, Institute of Pathology, University of Veterinary Medicine, Veterinaerplatz 1, 1210 Vienna, Austria; Sandra.Hoegler@vetmeduni.ac.at (S.H.); Rene.Brunthaler@vetmeduni.ac.at (R.B.); 4 Faculty of Veterinary Medicine, Institute of Virology, Justus-Liebig-University Giessen, Schubertstrasse 81, 35392 Giessen, Germany; Benjamin.J.Lamp@vetmeduni.uni-giessen.de

**Keywords:** emerging disease, Flaviviridae, pestivirus, atypical porcine pestivirus, Bungowannah virus, congenital tremor, Linda virus, novel Linda virus strain, viruses, Austria

## Abstract

Linda virus (LindaV) was first identified in a pig farm in Styria, Austria in 2015 and associated with congenital tremor (CT) type A-II in newborn piglets. Since then, only one more LindaV affected farm was retrospectively discovered 10 km away from the initially affected farm. Here, we report the recent outbreak of a novel LindaV strain in a farrow-to-finish farm in the federal state Carinthia, Austria. No connection between this farm and the previously affected farms could be discovered. The outbreak was characterized by severe CT cases in several litters and high preweaning mortality. A herd visit two months after the onset of clinical symptoms followed by a diagnostic workup revealed the presence of several viremic six-week-old nursery pigs. These animals shed large amounts of virus via feces and saliva, implying an important epidemiological role for within- and between-herd virus transmission. The novel LindaV strain was isolated and genetically characterized. The findings underline a low prevalence of LindaV in the Austrian pig population and highlight the threat when introduced into a pig herd. Furthermore, the results urge the need to better understand the routes of persistence and transmission of this enigmatic pestivirus in the pig population.

## 1. Introduction

The genus *Pestivirus* within the family *Flaviviridae* includes economically important pathogens of pigs. In addition to the OIE listed classical swine fever virus (CSFV, *Pestivirus C*) [1], Bungowannah virus (BungoV, *Pestivirus F*), atypical porcine pestivirus (APPV, *Pestivirus K*), and Linda virus (LindaV, *Pestivirus L*) have been detected in swine [2,3,4]. BungoV emerged in 2003 and is responsible for reproductive disorders, the birth of stillborn and mummified piglets, and sudden death in weaning age piglets [2]. A single outbreak of BungoV was detected in Australia and contained, but the virus has not yet been eradicated [5]. A spread of BungoV has been considered a threat to global porcine health, but there is no evidence to date that BungoV has established itself in other regions of the world [6,7,8,9]. In contrast, APPV occurs worldwide and is responsible for congenital tremor (CT) type A-II in piglets causing moderate economic losses [10,11,12,13,14].

LindaV was discovered as a novel “lateral-shaking inducing neurodegenerative agent” in a piglet-producing farm in Styria, Austria in 2015 [4]. Piglets infected in utero showed a severe lateral shaking of the whole body, causing incapability of sucking milk, which led to a high preweaning mortality [4]. CT type A-II was confirmed during histopathological examinations of diseased piglets with the presence of severe hypomyelination in the spinal cord and of viral antigen in tissues of the central nervous system (CNS) [4]. Experimental LindaV infections of immunocompetent piglets have been found to result in transient viremia and rapid seroconversion [15]. However, the virus persisted in lymphoid tissues and was still detectable after 21 days. To date, LindaV has been detected in the index case as well as in a farm 10 km away as a consequence of a nationwide screening for LindaV neutralizing antibodies [16]. This study revealed a very low seroprevalence of 0.15% (based on the number of sera screened) and 0.7% (at farm level) in Austria [16]. A novel, genetically closely related LindaV strain (LindaV strain Austria2) could be isolated from a serum sample in 2016 [16]. Interestingly, clinical signs of CT had never occurred in pigs of that farm, according to the farmer and responsible veterinarian.

In this study, we present the isolation of a novel LindaV strain (LindaV strain Austria3) causing clinically relevant disease in a farrow-to-finish farm in Carinthia, Austria in 2020/2021.

## 2. Materials and Methods

### 2.1. Farm Description

A commercial farrow-to-finish farm in Carinthia, Austria produces piglets with 60 Large White and Landrace crossbred sows in a continuous farrowing cycle. One Pietrain boar is used for semen production, natural insemination, and for sexual stimulation of sows, which are inseminated artificially. Piglets are weaned at 28 days of age. The piglets are routinely vaccinated against *Mycoplasma hyopneumoniae* and against porcine circovirus 2 (PCV2) using inactivated vaccines (*M. hyopneumoniae* in the first week of life with Suvaxyn^®^ MH-One, Zoetis Österreich GmbH, Vienna, Austria; PCV2 at 21 days of age with Suvaxyn Circo, Louvain-la-Neuve, Belgium).

Before onset of the reproductive disorders, new gilts were purchased in March 2020 from a commercial gilt producer and introduced into the herd after an isolation phase of eight weeks. Gilts and sows are immunized against parvovirosis and erysipelas using a combined inactivated vaccine (Parvoruvac, Ceva Santé Animale, Libourne, France) in the isolation unit and at the time point of weaning, respectively. Gilts and sows are vaccinated against porcine reproductive and respiratory syndrome virus (PRRSV) using a modified live virus vaccine (Porcilis PRRS, Intervet GesmbH, Vienna, Austria) in a five-month interval. Vaccination against swine influenza A virus using a trivalent vaccine was introduced after the reproductive disorders started.

### 2.2. Diagnostic Samples

The samples were obtained by veterinarians of the University Clinic for Swine (University of Veterinary Medicine, Vienna) for diagnostic purposes during a farm visit. Therefore, no ethical approval was needed for this study. A one-day-old non-viable piglet and an eight-week-old nursery pig with a paralysis of both hind legs were humanely euthanized under general anesthesia (anesthesia: 1.3 mg/kg azaperone and 10 mg/kg ketamine hydrochloride; euthanasia: T61 (5.0 mg/mL tetracaine hydrochloride, 50 mg/mL mebezonium iodide and 200 mg/mL embutramide; 1 mL/10 kg)). The following tissues were collected from the euthanized piglets: parotid gland, tonsil, lymph node (inguinal, tracheobronchial and mesenterial), heart, lung, liver, pancreas, duodenum, jejunum, ileum, caecum, colon, kidney, genital tract, urinary bladder, thymus, spleen, cerebellum, medulla oblongata, spinal ganglion, spinal cord, sciatic nerve, and umbilical cord (only newborn piglet). EDTA-treated blood and whole blood samples were drawn from two sows with previous CT litters, 15 nursery pigs and 2 euthanized piglets as mentioned above (euthanized eight-week-old nursery pig only EDTA-treated blood sample). Pooled fecal samples were collected from the gestation barn, farrowing crates, and the flat deck. Semen and a saliva swab sample were obtained from the boar. Further saliva swabs were collected from 12 sows and gilts and 15 nursery pigs (nursery pigs pooled swab sample). A pooled placenta sample from newly farrowed sows and tails from newborn piglets were also obtained.

Additionally, paraffin-embedded tissue samples (brain, liver, spleen, kidney, gastrointestinal tract, lung, and heart) originating from a CT affected piglet were kindly provided by the veterinary diagnostic laboratory of the federal state, Carinthia. These tissue samples were obtained during the acute congenital tremor phase in December 2020.

### 2.3. Pathological Examination and Immunohistochemistry

A full necropsy was performed on the eight-week-old nursery pig, euthanized because of a paralysis of both hind legs. Samples of brain, spinal cord, peripheral nerves, liver, spleen, kidneys, gastrointestinal tract, lung, lymph nodes, tonsils, thymus, pancreas, and salivary glands were fixed in 4% buffered formalin and embedded in paraffin wax. Two µm thick sections were cut and stained with hematoxylin and eosin (HE). Selected brain and spinal cord samples were stained with a combination of luxol fast blue and HE to assess the myelin content. Furthermore, paraffin-embedded samples (brain, liver, spleen, kidney, gastrointestinal tract, lung, and heart) from one CT affected piglet, which had been confirmed to be positive for LindaV by RT-PCR, were stained with HE.

CNS samples of both pigs were evaluated by immunohistochemistry using a primary anti-pestivirus E2 antibody (mouse monoclonal antibody (mAb) 6A5, dilution 1:100) for the detection of LindaV. Stainings were performed automatically on an autostainer (Lab Vision AS 360, Thermo Fisher Scientific, Waltham, MA, USA). Antigen retrieval was performed by pronase digestion. After application of the primary antibody, a polymer detection system (UltraVision LP Large Volume Detection System; Thermo Fisher Scientific, Waltham, MA, USA), consisting of a universal secondary antibody formulation conjugated to an enzyme-labeled polymer was used. The polymer complex was then visualized with diaminobenzidine (Labvision/Thermo Fisher Scientific, Waltham, MA, USA). Sections were counterstained with hematoxylin. Formalin fixed, paraffin-embedded spinal ganglia of pigs from the previous outbreak of LindaV in 2015 served as positive and negative controls, respectively.

### 2.4. Peripheral Blood Mononuclear Cell (PBMC) Isolation

EDTA-treated blood was centrifuged for 10 min at 2000× *g* and 4 °C. Plasma was collected from the supernatant and stored at −20 °C. The buffy coat layer was carefully removed (approximate volume 1 mL), transferred to a centrifuge tube containing 5 mL erythrocyte lysis buffer (Buffer EL, QIAGEN, Hilden, Germany) and thoroughly vortexed. The mixture was incubated for 10 min at 4 °C, followed by a centrifugation step for 10 min at 1000× *g* and 4 °C. The supernatant was removed and the procedure was repeated twice. Finally, the PBMC pellet was resuspended in 1 mL Dulbecco’s modified Eagle’s medium (DMEM, Biowest, Nuaillé, France).

### 2.5. Cell Culture

SK-6 cells [17] were cultured in DMEM (Biowest) supplemented with 10% heat-inactivated fetal calf serum (FCS, Corning, Tewksbury, MA, USA; tested negative for pestiviruses), 100 U/mL penicillin, and 100 µg/mL streptomycin. The cells were maintained at 37 °C and 5% CO_2_.

### 2.6. Indirect Immunofluorescence Assay

The indirect immunofluorescence assay was performed as previously described [4]. Briefly, SK-6 cells were fixed with 4% paraformaldehyde in PBS for 20 min at 4 °C and permeabilized with 1% Triton-X 100 (Merck, Darmstadt, Germany) in PBS for 5 min at room temperature. The cross-reactive mAb 6A5 (anti-BVDV E2) was used as a primary antibody and a goat anti-mouse IgG conjugated with Cy3 (Dianova, Hamburg, Germany) was used as a secondary antibody.

### 2.7. Virus Isolation

A total volume of 50 µL PBMCs resuspended in DMEM (as described in Section 2.4) was co-cultured with 5 × 10^4^ SK-6 cells on a 24-well cell culture plate (STARLAB, Hamburg, Germany). Cells and cell culture supernatant were passaged 72 h post co-cultivation. Successful infection of SK-6 cells was detected using an indirect immunofluorescence assay (as described in Section 2.6) and a LindaV-specific RT-qPCR assay (as described in Section 2.9).

### 2.8. Serum Virus Neutralization (SVN) Assay

The SVN assay was performed as previously described [16]. Briefly, sera were heat inactivated at 56 °C for 30 min and a five-fold serial dilution (1/5 starting dilution, 1/390,625 final dilution) was prepared in DMEM (Biowest) in a 96-well cell culture plate (STARLAB). An mCherry-labeled LindaV stock (1.78 × 10^5^ TCID_50_/mL) was diluted to 100 TCID_50_/50 µL, added to the serum dilutions and incubated for 2 h. A total number of 1 × 10^4^ SK-6 cells was added to the serum/virus-mixture and further incubated for 72 h. Serum controls, cell controls, positive and negative reference antisera, and a virus back titration were included. Cells were fixed with 4% paraformaldehyde and evaluated using a fluorescence microscope (Olympus IX70 fluorescence microscope; OLYMPUS, Hamburg, Germany).

The 50% neutralization dose (ND_50_/mL) was calculated based on the Spearman–Kaerber method and expressed as the reciprocal value (1/ND_50_/mL) of the serum dilution.

### 2.9. RNA Extraction and LindaV-Specific RT-qPCR Assay

Total RNA extraction was performed using the QIAamp Viral RNA Mini Kit (QIAGEN) according to the manufacturer’s instructions. A total volume of 140 µL serum, plasma, or semen was directly used for RNA extraction. Saliva swab samples were moistened with 1 mL PBS, mixed by vortexing, then were centrifuged and 140 µL supernatant was used for RNA extraction. Each tissue sample of 100 mg was mixed with 1 mL PBS in a 2 mL microcentrifuge tube containing stainless steel beads and homogenized at a frequency of 30/s for 3 min using a TissueLyser II (QIAGEN). After centrifugation, a volume of 140 µL homogenate supernatant was used for extraction. PBMCs were lysed by three freeze–thaw cycles, cell debris was removed by centrifugation, and 140 µL supernatant were used for RNA extraction.

For the detection of LindaV RNA, a LindaV-specific RT-qPCR assay was performed on a Rotor-Gene Q cycler (QIAGEN) using the Luna Universal Probe One-Step RT-qPCR Kit (NEB, Ipswich, Massachusetts, United States) as previously described [15]. A beta-actin RT-qPCR assay was used as an internal control, as previously described [16] under the same cycling conditions as the LindaV RT-qPCR assay. Saliva swab samples were spiked with a plasmid harboring the enhanced green fluorescent protein (EGFP) coding sequence prior to RNA extraction to exclude inhibitory factors in the RT-qPCR run, as the beta-actin RT-qPCR yields inconsistent results in the analysis of this sample material. The oligonucleotides EGFP-F (5′-GACCACTACCAGCAGAACAC-3′), EGFP-R (5′-GAACTCCAGCAGGACCATG-3′), and the probe EGFP-HEX (5‘-HEX-AGCACCCAGTCCGCCCTGAGCA-BHQ-1-3’) were used for the amplification of a 132 bp fragment of the EGFP sequence, as described by Hoffmann et al. [18], under the same cycling conditions as the LindaV RT-qPCR assay.

A ten-fold dilution series of a plasmid harboring the target sequence for the LindaV RT-qPCR assay was included in each run to obtain a standard curve for quantification of the viral load in the samples, as previously described [15]. Briefly, the copy number per reaction was estimated by the Rotor-Gene Q software (version 2.3.4.; QIAGEN) based on the copy number of the input plasmid DNA and converted to the respective genome equivalents of each sample (GE/mL, GE/g, or GE/swab). Since we used a DNA standard and not an RNA standard in our RT-qPCR assay, efficiency of the reverse transcription step could not be assessed, which could lead to minor deviations in the copy number calculations.

### 2.10. RT-PCRs, Sanger Sequencing and Sequence Analysis

A pan-pestivirus RT-PCR amplifying a fragment of the NS5B coding region was conducted using the OneTaq One-Step RT-PCR Kit (NEB) and oligonucleotides as previously described [4]. The following cycling conditions were used: 48 °C for 20 min, 94 °C for 1 min, followed by 45 cycles of 94 °C for 15 s, 50 °C for 30 s, 68 °C for 1:10 min, and a final elongation at 68 °C for 5 min. The amplicon with an approximate length of 800 bp was purified using the Quantum Prep PCR Kleen Spin Columns (Bio-Rad, Hercules, CA, USA) and the sequence of the purified PCR product was determined by Sanger sequencing (Eurofins Genomics, Ebersberg, Germany). The obtained sequence was analyzed using the Nucleotide Basic Local Alignment Search Tool (BLASTN) (https://blast.ncbi.nlm.nih.gov/Blast.cgi?PROGRAM=blastn&PAGE_TYPE=BlastSearch&LINK_LOC=blasthome, accessed on 4 June 2021).

The full genome of the novel LindaV strain Austria3 (GenBank accession number: OK086026) was obtained by a two-step RT-PCR approach with the generation of overlapping PCR fragments using oligonucleotides and cycling conditions as previously described [16]. The PCR products were purified using the Quantum Prep PCR Kleen Spin Columns (Bio-Rad) and the sequences were determined by Sanger sequencing (Eurofins Genomics). For the determination of the full-genomic sequence of the novel LindaV strain, a consensus sequence missing only the ultimate 5′- and 3′-ends was generated and compared to the available LindaV sequences (GenBank accession numbers: KY436034.1 (LindaV strain Austria1) and MZ027894.1 (LindaV strain Austria2)). Sequence analysis was performed using the DNA Strider 3.0 Software [19,20] and CLC Sequence Viewer 7.7.1 (CLC bio/QIAGEN Digital Insights, Aarhus, Denmark).

### 2.11. Phylogenetic Analysis

Phylogenetic analysis was performed using the CLC Sequence Viewer 7.7.1 (CLC bio/QIAGEN Digital Insights, Aarhus, Denmark) based on the complete genome sequences. The neighbor-joining algorithm and bootstrapping with 1000 replicates were used for the construction of the phylogenetic tree. The following pestivirus sequences were used for the analysis: Linda virus strain Austria1 (KY436034.1, *Pestivirus L*), Linda virus strain Austria2 (MZ027894.1), Linda virus strain Austria3 (OK086026), Bungowannah virus (EF100713.2, *Pestivirus F*), Dongyang pangolin pestivirus isolate DYAJ1 (MK636874.1, *Pestivirus P*), Phocoena pestivirus isolate NS170386 (MK910229.1, *Pestivirus M*), atypical porcine pestivirus 1 strain AUT-2016_C (KX778724.1, *Pestivirus K*), and classical swine fever virus strain Alfort_187 (X87939.1, *Pestivirus C*).

## 3. Results

### 3.1. Description of the Novel LindaV Outbreak

**Figure 1:**
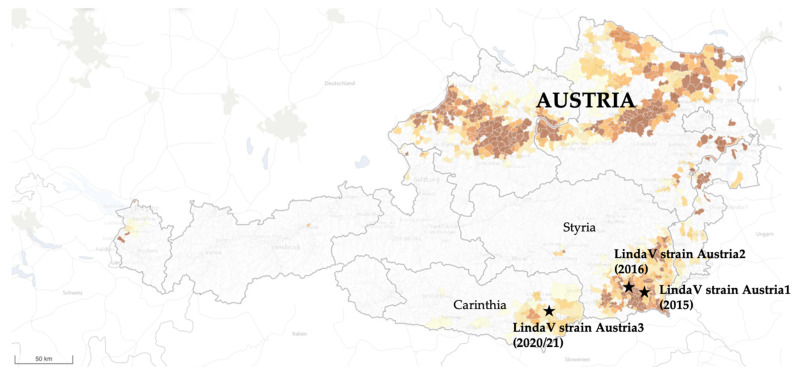
Locations of the farms where Linda virus (LindaV) strains Austria1, Austria2, and the novel strain Austria3 were isolated. Stars indicate the locations of the farms. Different brown shades mark regions with a high (dark brown) and a low (light brown) pig density. LindaV: Linda virus. (Modified from: https://www.statistik.at/atlas/?mapid=them_lw_as2010_viehbetriebe&layerid=layer1&sublayerid=sublayer0&languageid=0 (accessed on 18 August 2021). © Statistics Austria—Cartography and GIS, created 1 September 2018).

In autumn 2020, a farrow-to-finish farm in the federal state Carinthia in the south of Austria (Figure 1) reported cases of reproductive disorders (abortions, neonatal deaths, birth of stillborn and mummified piglets) in several sows and gilts. The symptoms started in October 2020, when four sows farrowed four weeks prior to the calculated farrowing date. These piglets were born alive, but died soon after birth. The subsequent seven litters presented with symptoms known for parvovirosis: mummies in different developmental stages, stillborn, and live-born piglets. The episode of reproductive failure was followed by the occurrence of severe CT in a total of 20 litters and associated with a high preweaning mortality of 80–90% in December 2020 (Video S1). The last CT affected litters occurred at the beginning of January 2021. During the CT episode in the farm, approximately 10–20% of piglets were born weak, and the return-to-estrus rate was 10%. The number of weaned piglets per sow and year dropped from an average of 28 to an average of 22 due to reproductive disorders and piglet mortality. Pooled organ samples from five CT affected piglets were sent to the University of Veterinary Medicine, Vienna in mid-January 2021. The analysis of the organ samples using a pan-pestivirus RT-PCR in the NS5B region yielded a positive result. In the diagnostic laboratory of the Institute of Virology at the University of Veterinary Medicine, Vienna, PRRSV and porcine parvovirus (PPV) were ruled out by PCR as differential diagnoses, and results of a PCV2 qPCR were below the level of quantification. Sequencing of the pestiviral NS5B amplicon with a length of approximately 800 bp and analysis of the consensus sequence using Nucleotide Basic Local Alignment Search Tool (BLASTN) revealed a sequence identity of 98.3% to the LindaV strain Austria1 found in 2015 (GenBank accession number: KY436034.1). The pooled organ samples were re-assayed using a LindaV-specific RT-qPCR in the 5′-UTR region, which determined a very high viral load of 3.9 × 10^8^ GE/g.

Subsequently, a farm visit was conducted in February 2021 for a diagnostic workup of the disease outbreak. At this time, no clinical symptoms associated with LindaV infection were observable and reproduction data approached pre-outbreak levels. Two sows, which farrowed the day before the farm visit, presented with homogenous litters of clinically healthy newborn piglets. All other sows and suckling piglets in the farrowing barn appeared clinically healthy. A batch of six-week-old nursery pigs surviving the CT phase presented as a heterogenous group with ill-thrift and runting piglets. An eight-week-old nursery pig suffering from a paralysis of both hind legs was observed and humanely euthanized. All sows and the boar appeared clinically healthy, whilst mild dry coughing was noticed in finisher pigs.

### 3.2. Diagnostic Workup

#### 3.2.1. Serum Virus Neutralization Assay

Sera of 15 six-week-old nursery pigs (P1–P15), plasma of an eight-week-old nursery pig, sera of two sows with previous CT litters, and serum of a one-day-old non-viable piglet were analyzed in the SVN assay (as described in Section 2.8).

**Figure 2:**
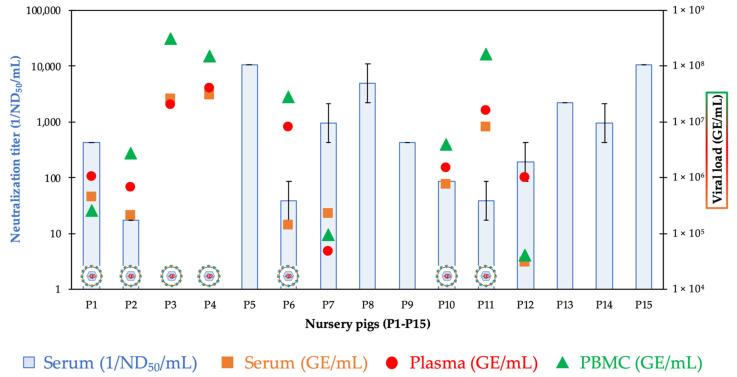
Results of the serum virus neutralization (SVN) assay (ND_50_/mL), the LindaV-specific RT-qPCR assay from serum, plasma and PBMCs (GE/mL), and virus isolation from PBMCs of six-week-old nursery pigs (P1–P15). Virus particles represent successful virus isolation through co-culturing of PBMCs with SK-6 cells. Neutralizing antibody titers are given as the reciprocal ND_50_ value and error bars indicate positive and negative standard deviations. ND_50_, 50% neutralization dose; GE, genome equivalents; PBMC, peripheral blood mononuclear cell.

High neutralizing antibody titers ≥1/193.2 ND_50_/mL were detectable in nine nursery pigs (P1, P5, P7–P9, and P12–P15). Intermediate to low neutralizing antibody titers between 1/17.2 ND_50_/mL and 1/86.4 ND_50_/mL were found in four animals (P2, P6, P10, and P11). Two nursery pigs (P3 and P4) did not show any neutralizing activity (Figure 2). A high neutralizing activity of 1/10,640 ND_50_/mL was determined in the plasma of the eight-week-old nursery pig, euthanized because of a paralysis of both hind legs. Two sows with previous CT litters showed a strong neutralizing activity against LindaV (both 1/2180 ND_50_/mL). No neutralizing activity was detectable in a one-day-old, non-viable piglet.

#### 3.2.2. LindaV-Specific RT-qPCR Assay

The LindaV-specific RT-qPCR assay and the RT-qPCR assays for the internal control genes beta-actin and eGFP were performed as described in Section 2.9. All samples analyzed in the LindaV RT-qPCR assay were positive in the RT-qPCR assays for the internal control genes beta-actin and eGFP.

The results of the LindaV-specific RT-qPCR assay of the six-week-old nursery pigs (P1–P15; serum, plasma, and PBMCs) are shown in Figure 2 and described in detail in Supplementary Table S1. The analysis of a pooled saliva swab sample and a pooled fecal sample from the six-week-old nursery pigs (P1–P15) confirmed viral shedding, showing a high viral load of 1.17 × 10^6^ GE/swab (pooled saliva swab sample) and 2.65 × 10^6^ GE/g (pooled fecal sample) (Supplementary Table S1).

Furthermore, tissue samples from different organs and blood samples (plasma and PBMCs) of an eight-week-old nursery pig, euthanized because of a paralysis of both hind legs, were analyzed in the RT-qPCR assay. LindaV RNA was detected in lymphoid organs (inguinal lymph node and tonsil) and tissues of the CNS (cerebellum and medulla oblongata) and the peripheral nervous system (PNS; spinal ganglion). The viral loads ranged from 2.25 × 10^5^ GE/g to 5.36 × 10^6^ GE/g (Supplementary Table S2). All other tissue samples tested negative by RT-qPCR and viremia was not detectable in this animal.

LindaV RNA was not detectable in any of the samples obtained from sows and gilts (feces, saliva swab samples, and blood samples). All samples obtained from the boar, the newborn piglets, and the pooled placenta sample were negative in the LindaV-specific RT-qPCR.

#### 3.2.3. Virus Isolation

Virus isolation was conducted through the co-culturing of PBMCs with susceptible SK-6 cells (as described in Section 2.7). Virus isolation was successful in 7 out of 15 nursery pigs (P1–P4, P6, P10, and P11) (Figure 2 and Supplementary Table S1). No virus could be isolated from PBMCs of the sows and the euthanized one-day-old piglet.

#### 3.2.4. Histopathology and Immunohistochemistry

**Figure 3:**
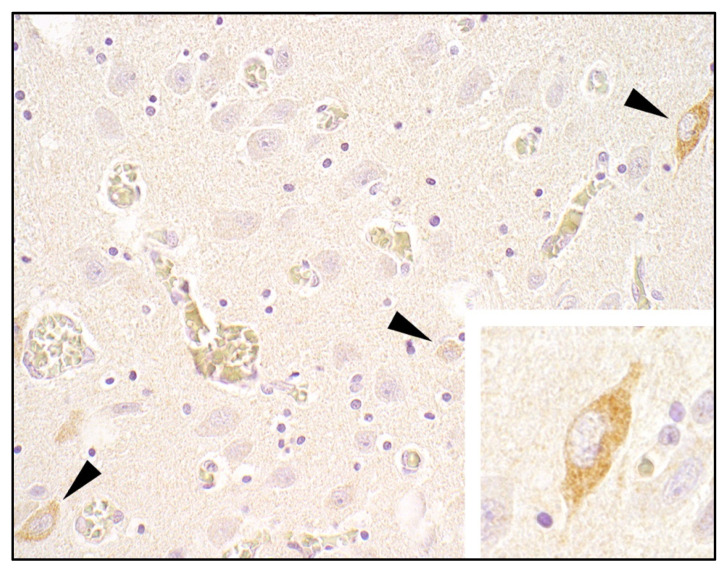
Detection of LindaV by immunohistochemistry in the cytoplasm of neurons (arrowheads) in the brainstem of a CT piglet. Primary antibody: pestivirus E2-specific monoclonal antibody 6A5; counterstain: hematoxylin, magnification 400×, insert 600×.

Paraffin-embedded samples of cerebellum and brainstem from one CT piglet were available for histological and immunohistochemical evaluation (as described in Section 2.3). HE staining showed no lesions, but LindaV could be detected in the cytoplasm of neurons by immunohistochemistry (Figure 3).

Furthermore, tissue samples of the eight-week-old nursery pig were analyzed. Immunohistochemistry for the detection of LindaV showed no positive results in any of the tissues. While determining cause of the paralysis of both hind legs, randomly distributed foci of neuronal necrosis in the gray matter and degeneration of the white matter of the caudal cervical spinal cord with very mild reactive changes and scattered small hemorrhages were detected. The etiology of these lesions could not be revealed. Multifocal scattered perivascular lymphoplasmacellular infiltrations were present in the brain and spinal cord.

### 3.3. Genetic Characterization of the Novel LindaV Strain Austria3

**Figure 4:**
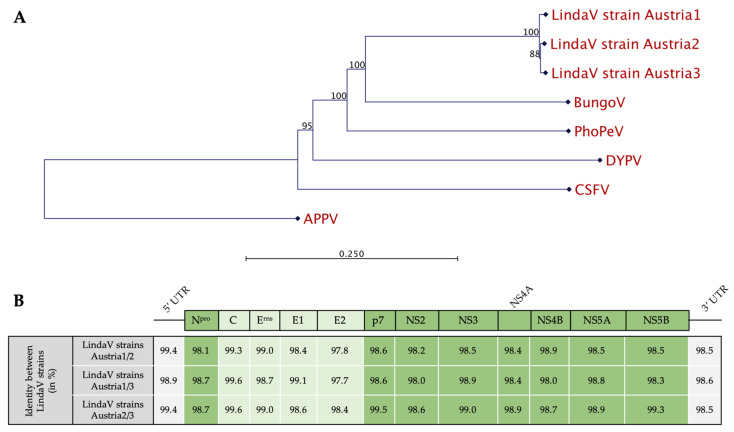
Phylogenetic analysis of selected pestivirus species and sequence analysis of the full-genomic sequences of the so far known Linda virus strains. (**A**) An unrooted phylogenetic tree was constructed based on the full-genomic sequences of LindaV strain Austria1, LindaV strain Austria2, LindaV strain Austria3, Bungowannah virus, Phocoena pestivirus isolate NS170386, Dongyang pangolin pestivirus isolate DYAJ, CSFV strain Alfort_187, and APPV strain AUT-2016_C using the neighbor-joining algorithm and bootstrapping with 1000 replicates. Bootstrap values are shown in percentage at each node. (**B**) Sequence identities between the different coding regions of the viral genome of LindaV strains Austria1, Austria2, and Austria3 are given in percentage. The coding regions for the nonstructural proteins are highlighted in dark green and coding regions for the structural proteins are in light green. DYPV, Dongyang pangolin pestivirus; PhoPeV, Phocoena pestivirus; BungoV, Bungowannah virus; APPV, atypical porcine pestivirus; CSFV, classical swine fever virus; UTR, untranslated region; C, Core; E, envelope glycoprotein; NS, nonstructural protein.

The full genome sequence of the novel LindaV strain Austria3 (OK086026) was obtained using a two-step RT-PCR approach and Sanger sequencing. We chose the sample with the highest viral load in the RT-qPCR assay (3.16 × 10^8^ GE/mL; PBMCs of nursery pig P3) for this sequencing attempt. A consensus sequence of 12,568 nt was determined and compared with the sequences of LindaV strain Austria1 (KY436034.1) and LindaV strain Austria2 (MZ027894.1) (Figure 4). Interestingly, LindaV strain Austria2 and LindaV strain Austria3 were found to be slightly more closely related than they were to the index case LindaV strain Austria1; the total nucleic acid sequence identity was 98.9% compared to 98.5%, respectively. The sequence identities between the different coding regions of the LindaV genome are shown in Figure 4B. The divergence in the nucleic acid sequence resulted in a comparable total amino acid identity of 98.3% between both LindaV strain Austria1 and LindaV strain Austria2 as well as LindaV strain Austria1 and LindaV strain Austria3. In contrast, total amino acid identity was 99.1% when comparing LindaV strain Austria2 and LindaV strain Austria3.

## 4. Discussion

The discovery of LindaV in Styria, Austria in 2015 expanded the group of pestiviruses infecting the host species swine and revealed the long-sought European relative to BungoV from Australia [4]. Despite an elevated level of attention after the first LindaV outbreak, neither signs of viral spread nor the existence of this virus in other pig herds have been observed [8,12]. Nevertheless, LindaV was found in a farm a 10 km distance from the index case by retrospective sero-surveillance without clinical indications [16]. In this paper, we describe a new outbreak of LindaV that occurred six years later in another province of Austria. Similar to the index case, CT and high preweaning mortality occurred in the piglets following reproductive disorders in the sows. Furthermore, LindaV was detectable in six-week-old pigs, which suffered from CT as newborn piglets, suggesting a chronic or persistent infection in those animals.

Intrauterine infections with pestiviruses at early stages of gestation may cause immunotolerance [21,22,23,24,25]. Immunotolerant animals become persistently infected (PI) and shed large amounts of virus throughout their lifespan. After intrauterine infection of the fetus with BungoV, a long-lasting, high-level viremia above 10^6^ GE/mL up to 75 days of age has been observed [25]. While studies assessing the outcome of fetal infection with LindaV are still missing, the high-level viremia of up to 3 × 10^8^ GE/mL together with the lack of humoral immune response against LindaV in individual animals (piglets P3 and P4) indicate the occurrence of such chronically, possibly persistently infected animals. Furthermore, these six-week-old animals shed large amounts of virus via the fecal and oral route, supporting this hypothesis.

High titers of neutralizing antibodies were found in some viremic piglets, which could have been passively acquired by colostrum ingestion. Unfortunately, no discrimination between maternally-derived and actively acquired antibodies was possible in our test. Titers of maternally-derived antibodies can last for a long time after pestiviral infection. Maternally-derived antibodies against BVDV have been detected around 190 days [26] and against CSFV over a period of seven weeks [27]. Therefore, the presence of maternal antibodies cannot be excluded.

It has been shown that PBMCs represent a reliable specimen for the detection of the cell-associated viremia of BVDV [28] and CSFV [29]. In this study, we detected high viral loads of LindaV RNA in PBMCs and isolated the virus through co-culturing with SK-6 cells. Several highly viremic animals showed a 10-fold higher viral load in PBMCs compared to serum and plasma, suggesting that the analysis of PBMCs represents a more sensitive detection method for the viremic phase of LindaV infections. Virus isolation from PBMCs was successful starting from 2.58 × 10^5^ GE/mL, even though a high neutralizing antibody titer of 1/434 ND_50_/mL was observed in one animal (P1). In this case, virus isolation attempts from serum or plasma could lead to false negative results due to circulating neutralizing antibodies.

In the eight-week-old nursery pig suffering from a paralysis of both hind legs, focally extensive acute lesions in gray and white matter in the caudal cervical spinal cord were present with only mild reaction. Neither the distribution nor the type of lesions were representative of a viral infection. Therefore, the lesions were thought to be most likely of ischemic or traumatic origin. Unrelated to these lesions, multifocal scattered perivascular lymphoplasmacellular infiltrations were detected in the brain and spinal cord, which might be due to an infection with LindaV, which is in accordance with the viral RNA detected in structures of the CNS by RT-qPCR. The negative results in the immunohistochemical analysis could indicate a lower sensitivity of this technique compared to the RT-qPCR assay.

There is high sequence identity between all three currently known LindaV strains (Austria1–Austria3). We found no evidence for transmission or epidemiological links between the cases in Styria and the new outbreak in Carinthia. Therefore, we must assume a low but steady prevalence of LindaV in Southern Austria. The purchase of new gilts from a commercial gilt producer and the disease outbreak approximately six months after their introduction into the pig herd may be a possible route of transmission. Investigations of the presence of LindaV in the gilt-producing facility are underway. However, gilt-producing farms in Austria usually sell pigs to several pig holdings and a single disease outbreak would be unlikely considering the low seroprevalence of LindaV infections in the Austrian pig population [16]. Since our seroprevalence study could not detect reservoirs in domestic pig herds, it is reasonable to postulate a reservoir host in the wild. Wild boar and wild ruminant species could represent the reservoir of LindaV. Because pestiviruses have also been described in the recent past in some non-cloven-hoofed animals [30,31,32,33], other wildlife reservoirs, such as rodents or bats, cannot be excluded. To prevent further introduction of the virus into the domestic pig population, identification of reservoir hosts is critical. A steady surveillance should also be established in the risk region of Southern Austria to prevent spread via clinically inconspicuous, long-term viremic animals.

## 5. Patents

The authors B.L., L.S., and T.R. are inventors of a patent on LindaV pestivirus (PCT/EP2017/084453; Isolation of a novel pestivirus causing congenital tremor).

## Data Availability

All data analyzed or generated during this study are included in the manuscript.

## Supplementary Materials

The following supporting information can be downloaded at: https://www.mdpi.com/article/10.3390/v14020326/s1, Video S1: Piglets affected with congenital tremor (CT), Supplementary Table S1: Results of serum virus neutralization (SVN) assay (1/ND_50_/mL), RT-qPCR assay (GE/mL; GE/g; GE/swab) and virus isolation from six-week-old nursery pigs, Supplementary Table S2: Results of SVN assay (1/ND_50_/mL), RT-qPCR assay (GE/mL, GE/g) and virus isolation from an eight-week-old euthanized nursery pig suffering from a paralysis of both hind legs.
