# Supplementary material for: New Emergence of the Novel Pestivirus Linda Virus in a Pig Farm in Carinthia, Austria
Source: Viruses. 2022 Feb 5;14(2):326. doi: 10.3390/v14020326 (PMC8874435; doi:10.3390/v14020326)
Supplement: Supplementary file 1 [file viruses-14-00326-s001.zip › Supplementary Tables.pdf]

Supplementary Table S1. Results of serum virus neutralization (SVN) assay (1/ND_{50}/mL), RT-qPCR assay (GE/mL; GE/g; GE/swab) and virus isolation from six-week-old nursery pigs. ND_{50}, 50% neutralization dose; GE, genome equivalents; PBMC, peripheral blood mononuclear cell.

	Animal ID	1/ND_{50}/mL	Serum GE/mL	Plasma GE/mL	PBMC GE/mL[1]	PBMC Virus Isolation	Feces GE/g	Saliva GE/swab
Six-week-old nursery pigs (P1-P15)	P1	1/434	4.46×10^5	1.04×10^6	2.58×10^5	+		
	P2	1/17.2	2.08×10^5	6.69×10^5	2.81×10^6	+		
	P3	neg.	2.62×10^7	2.03×10^7	3.16×10^8	+		
	P4	neg.	3.04×10^7	4.09×10^7	1.52×10^8	+		
	P5	1/10,640	neg.	neg.	neg.	-		
	P6	1/38.4	1.42×10^5	8.19×10^6	2.87×10^7	+		
	P7	1/968	2.26×10^5	4.80×10^4	9.61×10^4	-		
	P8	1/4,860	neg.	neg.	neg.	-		
	P9	1/434	neg.	neg.	neg.	-		
	P10	1/86.4	7.55×10^5	1.49×10^6	3.95×10^6	+		
	P11	1/38.4	8.15×10^6	1.63×10^7	1.66×10^8	+		
	P12	1/193.2	3.08×10^4	1.02×10^6	4.10×10^4	-		
	P13	1/2,180	neg.	neg.	neg.	-		
	P14	1/968	neg.	neg.	neg.	-		
	P15	1/10,640	neg.	neg.	neg.	-		
	P1-P15 (pooled sample)						2.65×10^6	1.17×10^6

[1] GE/mL PBMC pellet resuspended in 1 mL DMEM

Supplementary Table S2. Results of SVN assay (1/ND_{50}/mL), RT-qPCR assay (GE/mL, GE/g) and virus isolation from an eight-week-old euthanized nursery pig suffering from a paralysis of both hind legs. ND_{50}, 50% neutralization dose; GE, genome equivalents; PBMC, peripheral blood mononuclear cell.

**Animal ID**	**1/ND_{50}/ mL**	**Plasma GE/mL**	**PBMC GE/mL**[1]	**PBMC Virus Isolation**	**Tissue samples (GE/g; only positive results shown)**				
					**inguinal lymph node**	**medulla oblongata**	**tonsil**	**spinal ganglion**	**cerebellum**
Euthanized nursery pig (eight-week-old)	1/10,640	neg.	neg.	-	2.28×10^6	2.25×10^5	2.56×10^6	5.36×10^6	1.19×10^6

[1] GE/mL PBMC pellet resuspended in 1 mL DMEM
